# Internet Usage by Polish Patients With Multiple Sclerosis: A Multicenter Questionnaire Study

**DOI:** 10.2196/11146

**Published:** 2019-02-01

**Authors:** Andrzej Potemkowski, Waldemar Brola, Anna Ratajczak, Marcin Ratajczak, Jacek Zaborski, Elżbieta Jasińska, Anna Pokryszko-Dragan, Ewa Gruszka, Marta Dubik-Jezierzańska, Aleksandra Podlecka-Piętowska, Monika Nojszewska, Krystyna Gospodarczyk-Szot, Adam Stępień, Katarzyna Gocyła-Dudar, Marzena Maciągowska-Terela, Jacek Wencel, Radosław Kaźmierski, Alina Kułakowska, Katarzyna Kapica-Topczewska, Witold Pawełczak, Halina Bartosik-Psujek

**Affiliations:** 1 Department of Clinical Psychology and Psychoprophylaxis University of Szczecin Szczecin Poland; 2 Institute of Physiotherapy Faculty of Medicine and Health Sciences Jan Kochanowski University Kielce Poland; 3 Pomeranian Medical University Szczecin Poland; 4 Clinical Trial Centre EuroMedis Szczecin Poland; 5 Department of Neurology Specialist Hospital in Międzylesie Warsaw Poland; 6 Clinical Center RESMEDICA Kielce Poland; 7 Department of Neurology Wroclaw Medical University Wroclaw Poland; 8 Outpatient MS Clinic Lower Silesian Specialistic Hospital Wroclaw Poland; 9 Department of Neurology Medical University of Warsaw Warsaw Poland; 10 Department of Neurology Military Institute of Medicine Warsaw Poland; 11 Department of Neurology and Cerebrovascular Disorders L Bierkowski Hospital Poznan University of Medical Sciences Poznan Poland; 12 Department of Neurology Medical University of Białystok Białystok Poland; 13 Department of Neurology Specialist Hospital Kalisz Poland; 14 Medical Faculty University of Rzeszów Rzeszów Poland

**Keywords:** multiple sclerosis, internet, information seeking, doctor-patient relationship

## Abstract

**Background:**

The internet is a source of knowledge and medium widely used in services that facilitate access to information and networking. Multiple sclerosis (MS) patients find the possibility of acquiring information relating to their condition particularly rewarding.

**Objective:**

We aimed to identify Polish MS patients’ preferences by analyzing a percentage of internet users and determining the most common search subjects and patients’ approach to information on the internet. Disability connected with the condition, its duration, and other factors that influence patients’ internet use were examined along with instances of relations established through the internet and their durability.

**Methods:**

The study examined 1045 patients (731 women, 314 men) treated in 10 Polish MS centers, of whom 932 (89.19%) declared to be internet users. Their average age was 40.65 (SD 11.06) and average MS duration was 9.08 (SD 6.97) years. The study used a proprietary survey on information seeking, the range of searched subjects, and internet usage frequency.

**Results:**

The majority of the patients (494/932, 53.0%) used the internet 6-7 times per week and 4.3% (40/932) declared they spent minimum 2 hours per day. The most commonly searched subjects were world news (604/932, 72.9% of patients using the internet); 60.8% (504/932) searched for information on their condition, particularly for new treatment methods (562/932, 67.8%) and the course of illness (520/932, 62.7%). One’s sex had no impact on internet usage (female vs male, odds ratio [OR] 1.13, 95% CI 0.72-1.77), although a patient’s age might, at varying degrees. We found several significant associations using a .05 significance level: a patient with higher education used the internet 9 times more often than one with primary education (OR 8.64, 95% CI 3.31-22.57); lasting relationships increased chances of internet usage by 10-fold compared to widowers (OR 0.12, 95% CI 0.05-0.31); living in a city with a population over 100,000 increased chances by nearly 6 times compared with the countryside (OR 5.59, 95% CI 2.72-11.48); the relapsing-remitting MS type saw a 2-fold increase compared with the primary progressive MS type (OR 0.47, 95% CI 0.29-0.75); and those needing assistance were 2 times less likely to use the internet than patients who could move independently (OR 0.53, 95% CI 0.31-0.89). More than half of the patients (489/932, 52.5%) did not discuss the information found on the internet with their neurologists; 15.9% (148/932) believed that relationships established through the internet can be stable.

**Conclusions:**

The majority of Polish patients use the internet as a crucial information source on their condition and innovative treatment methods. The internet can be helpful in establishing new relationships, which are usually short-lived. Polish patients do not frequently discuss the information gathered on the internet with their doctors.

## Introduction

The internet is one of the most popular sources for information and entertainment. Multiple sclerosis (MS) makes day-to-day functioning difficult because it limits interpersonal relationships and presence in societal life. Communication and exchange of information, ideas, and feelings’ grow more and more troublesome [1]. Studies on direct verbal communication show that almost half of the examined participants (46%) claimed poor or very poor ability to undertake social roles. Half of the participants claimed they were unhappy with their interpersonal activity [1]. Communication problems indeed influence restrictions in many social roles common in adult life, including work, home management, and leisure activities. Yorkston et al identified 5 variables (cognitive skills, speech severity, speech usage, physical activity, and education) that were the most important predictors of communication participation [1]. These restrictions would seem to be an important target for intervention.

Some studies reveal that internet use leads to deepened psychopathology, alienation, and loneliness, but those results are ambiguous. In their longitudinal studies of 1998, Kraut et al initially claimed that using the internet contributed to one’s sense of loneliness and higher depression rates, but when the same trial was repeated in 2002, it failed to confirm previous conclusions [2,3].

The subject of patients’ condition is of particular interest to them; the amount and incomprehensibility of medical terminology and the uncertainty connected with the clinical course of the disease all serve as motivation for further research on the internet. From the very time the internet became widely accessible, scientists have conducted studies on human behavior, the influence of the internet on one’s life and the correlations between its usage and one’s living standards and psychical and societal life, as well as those suffering from MS [4,5,6,7].

Internet communication may happen synchronously or asynchronously. Forum users may be online at the same time and communicate verbally on a given subject, having relatively high control over their answer and the ability to edit it if needed [8].

From a psychological point of view, verbal communication, with sounds and vision, is preferred. It is important for those suffering from MS as a supporting tool or even in psychological therapy [9,10]. Nevertheless, text dialogue grants more comfort to those having difficulties with communication because it provides anonymity and allows one to stop the contact at any given moment [11]. Atreja et al indicated that MS patients typically search for information right before or after a doctor’s appointment and use the information available online to check medical terminology [12]. Hay et al showed that majority of MS patients surfed the internet before their first appointment [13]. Even though the information found on the internet cannot replace that provided by a doctor, two-thirds of patients were reluctant to share the information gained independently with their doctors—which suggests that patients fear that their research could be understood as a lack of trust toward their doctors [13]. The quality and content found online and available to MS patients were assessed as varied, although some websites did offer almost all crucial information [14].

The studies also focused on personal traits, language barriers, and the impact other illnesses have on societal life [15-18]. The results indicated that the internet might have a positive impact on establishing new contacts, especially in the case of patients struggling with their symptoms. In high neuroticism, a limited amount of stimuli proved to be helpful in communication and relationship building [15]. Establishing and maintaining new relations constitutes one of the most significant psycho-social needs of MS patients that can be met through internet usage [18-20].

The aim of our study was to determine the percentage of Polish MS patients using the internet, the most commonly searched subjects, patients’ approach to the information found on the internet, and assess the impact of the condition’s duration, different levels of disability, and other demographic factors on the frequency and durability of relationships established online.

## Methods

### Design

We carried out the assessment in 2 stages: (1) the pilot study (drafting and verification of the questionnaire) and (2) the survey proper. The first stage (September-December 2015) consisted of a pilot examination in a group of 83 MS patients who declared to be using the internet; its aim was to evaluate the research tool developed for the study [18]. The questionnaire was developed by the authors and based on their experiences. The data collection was carried out at the Department of Clinical Psychology and Psychoprophylaxis of the University of Szczecin in Szczecin. The second stage included the proper survey conducted in 10 centers of MS diagnosis and treatment in 7 Voivodeships of Poland (8 clinical wards and outpatient MS clinics, an MS rehabilitation center, and a center for clinical trials related to MS). An anonymous questionnaire containing 11 questions on demographic data and 14 connected to one’s internet usage was filled out by patients during their appointments.

### Setting and Participants

Participants were recruited between February 2016 and December 2016 for the cross-sectional study. Patients were invited to fill out a paper version of the survey. The study included patients over the age of 18 years suffering from MS (clinically defined according to the McDonald criteria, 2010). Informed consent was obtained from all individual participants included in the original clinical studies.

### Statistical Analysis

All the continuous variables were verified owing to normal distribution with the Kolmogorov-Smirnov test. They were described using means, SDs, medians, quartiles, and minimal and maximal values. Verification of statistical differences between 2 groups was done using the Student *t* test and Mann-Whitney *U* test. For many groups, the analysis of variance and Kruskal-Wallis tests were used. Discontinuous variables were described using the quantity and frequency of occurrence. For studying statistical relations among discontinuous variables, the Pearson chi-square test or the Fisher exact test were used. For studying correlations among discontinuous variables, sequential and nominal (dummy variables: 0/1) and continuous variables the Pearson or Spearman rank correlations were used. The results were described using the correlation coefficient *r* and probability *P*. For the Pearson correlation, the regression line equations were also provided. A *P* value of <.05 was considered statistically significant. Data were analyzed using SPSS software, version 17.0 (2008; SPSS Inc, Chicago, IL, USA).

### Ethics Approval

The study received ethics approval from the Department of Psychology, University of Szczecin Ethics Committee (#17/2015), and all participants received explanations of the study objectives and signed informed consents.

## Results

The study involved 1045 people (731 female and 314 male) with an average age of 40.65 (SD 11.06) years. The average MS duration was 9.08 (SD 6.97) years (Table 1). The majority of participants (89.19%) used the internet (932/1045 patients: 652 females and 280 males); only 10.81% (113/1045 patients) did not use the internet. In the internet user group, 53.0% (494/932) stated they used the internet more than 4-5 times a week, and 56.4% (526/932) of the patients spent an average of 1-4 hours per week browsing the internet. Only 4.7% (44/932) of the patients claimed to use the internet more than 14 hours per week (at least 2 hours per day; Table 2).

Subjects that were most commonly searched were world news (604/932, 72.9% of patients using the internet); 60.8% (504/932) searched for information on their disease and 54.5% (452/932) looked for information on treatment. Over half of the patients examined (423/932, 51.0%) communicated with others via the internet. Only 1.8% (15/932) used it to search for job offers (Table 3).

Nearly 15% (120/932) of participants used the internet to establish new relationships and find new communities of MS patients. Relationships developed through the internet were deemed to be unstable by 32.9% (307/932) of patients and durable by 15.9% (148/932); 28.8% (268/932) of the examined patients sustained such relations. Only 13.2% (123/932) reported meeting other MS patients online.

While searching for information on MS, 67.8% (562/932) of the patients were looking for data on innovative treatment methods; 62.7% (520/932) focused on the course of the disease and 41.9% (347/932) on estimates concerning lifespan and diagnostic methods (Table 4).

The study showed that internet usage does not depend on sex (female vs male, odds ratio [OR] 1.13, 95% CI 0.72-1.77), although it might depend on age (Table 5).

**Table 1:** Demographic and clinical characteristics of study participants (N=1045).

Characteristics		MS^a^ internet users (n=932)	MS internet nonusers (n=113)	*P* value
**Patients invited, n (%)**		932 (89.18)	113 (10.81)	<.001
	Female	652 (62.39)	79 (7.55)	.99
	Male	280 (26.79)	34 (3.25)	
**Age (years), mean (SD)**		40.51 (10.94)	41.84 (12.05)	.23
	Female	40.64 (11.12)	41.24 (12.38)	.6
	Male	40.24 (10.54)	43.22 (11.29)	.12
**Disease duration (years), mean (SD)**		8.99 (6.86)	9.81 (7.87)	.24
	Female	9.13 (7.00)	10.08 (7.82)	.26
	Male	8.62(6.69)	9.17 (8.01)	.66
**Education, n (%)**				<.001
	Primary	36 (3.9)	8 (7.1)	
	Secondary	358 (38.4)	34 (30.1)	
	Higher	409 (43.9)	32 (28.3)	
	Vocational	129 (13.9)	39 (34.5)	
**Marital status, n (%)**				.01
	Married	612 (65.7)	73 (64.6)	
	Divorced	84 (9.0)	11 (9.7)	
	Unmarried	217 (23.3)	21 (18.6)	
	Widow or widower	19 (2.0)	8 (7.1)	
**Employment, n (%)**				.003
	Full-time	508 (54.4)	55 (48.7)	
	Part-time	65 (6.9)	6 (5.3)	
	Pension	300 (32.2)	34 (30.1)	
	Never worked	59 (6.4)	18 (15.9)	
**Residence, n (%)**				.29
	Countryside	361 (38.7)	44 (38.9)	
	City of up to 10,000	145 (15.6)	17 (15.0)	
	City of up to 100,000	165 (17.7)	13 (11.5)	
	City of over 100,000	261 (28.0)	39 (34.5)	
**Living with, n (%)**				.3
	Partner and children	467 (50.1)	56 (49.6)	
	Partner	154 (16.5)	18 (15.9)	
	Children	50 (5.4)	5 (4.4)	
	Parents	162 (17.4)	15 (13.3)	
	Alone	99 (10.6)	19 (16.8)	
**MS type, n (%)**				.80
	Relapsing-remitting multiple sclerosis	360 (38.6)	45 (39.8)	
	Secondary progressive multiple sclerosis	348 (37.3)	44 (38.9)	
	Primary progressive multiple sclerosis	224 (24.0)	24 (21.2)	
**Mobility, n (%)**				.88
	Without assistance	736 (79.0)	90 (79.7)	
	Independently, with orthopedic device	135 (14.5)	17 (15.0)	
	With assistance	61 (6.6)	6 (5.3)	

^a^MS: multiple sclerosis.

**Table 2:** Internet use by patients with multiple sclerosis (n=932).

Internet use		Patients, n (%)
**Frequency of browsing websites on the internet**		
	Once a week	78 (9.4)
	2-3 times a week	120 (12.9)
	4-5 times a week	130 (13.9
	More often	494 (53.0)
	Difficult to determine	110 (11.8)
**Hours on the internet per week**		
	1-4	526 (56.4)
	5-7	182 (19.5)
	8-14	57 (6.1)
	More	44 (4.7)
	Difficult to determine	123 (13.2)

**Table 3:** Internet services used by Polish patients with multiple sclerosis (n=932).

Service	Patients, n (%)
World news	604 (72.9)
MS^a^ information	504 (60.8)
Shopping	458 (55.6)
Treatment information	452 (54.5)
Communication	423 (51.0)
Entertainment, movies	408 (49.2)
New acquaintances	120 (14.5)
Meeting people and communities with MS	117 (14.1)
Other—job seeking	15 (1.8)
Other—scientific research	11 (1.3)

^a^MS: multiple sclerosis.

**Table 4:** Type of health information sought about multiple sclerosis at the time of most recent search (n=932).

Type of health information sought	Patients, n (%)
Innovative treatment methods	562 (67.8)
Course of MS^a^	520 (62.7)
Medication reviews (efficiency)	407 (49.1)
Diagnostic methods in MS	347 (41.86)
Prognosis and lifespan	347 (41.9)
MS treatment costs	336 (40.5)
MS diagnosis—criteria	324 (39.1)
New medication—analysis	285 (34.4)
MS treatment centers’ reviews	238 (28.7)
MS doctors’ reviews	196 (23.6)
Stem cells treatment—results	192 (23.2)
Alternative methods—reviews	184 (22.2)
MS and pregnancy	158 (19.1)
Treatment center using stem cells	158 (19.1)
MS and marriage	104 (12.6)
MS and sexuality	97 (11.7)
Raising children	96 (11.6)
MS and chronic venous insufficiency (results)	54 (6.5)
MS and diet	5 (0.6)

^a^MS: multiple sclerosis.

Higher education was associated with almost a 9-fold increase in internet usage compared with primary education (OR 8.64, 95% CI 3.31-22.57) and widows or widowers tended to use it 10 times less often than those in a partner relationship (OR 0.12, 95% CI 0.05-0.31). Living in a city with a population greater than 100,000 increased the chances 6-fold compared with living in the countryside (OR 5.59, 95% CI 2.72-11.48). Patients requiring assistance were 2 times less likely to use the internet compared with those that were able to move independently (OR 0.53, 95% CI 0.31-0.89) and patients with relapsing-remitting MS were 2 times less likely to use the internet than those suffering from primary progressive MS (OR 0.47, 95% CI 0.29-0.75). A stable course of condition (with no relapses) increased the chances by 1.5 times compared with relapsing-remitting MS (OR 1.50, 95% CI 0.87-2.57).

Over half of the participants (489/932, 52.5%) did not discuss the information on MS found on the internet with their neurologists. Moreover, 75.2% (676/932) recommend other patients to use the internet, but 82.4% (768/932) warned about the application of data about MS found online. The study also evaluated the credibility of the websites according to the patients (0: unreliable and 10: reliable). The participants of this part of the study decided that the most reliable websites belonged to the MS communities and other patients suffering from MS, that is, blogs (Table 6).

**Table 5:** Internet usage according to sociodemographic and health-related factors.

Categories			Odds ratio		95% CI		*P* value
Sex: men vs women		1.13		0.72-1.77		.59	
**Age (years)**							
	33-48 vs <33	0.31		0.13-0.75		.01	
	>48 vs <33	0.08		0.03-0.18		<.001	
**Duration MS^a^** **(years)**							
	4-12 vs <4	1.09		0.62-1.91		.78	
	>12 vs <4	0.48		0.27-0.87		<.001	
**Education**							
	Secondary vs primary	1.94		0.83-4.51		.13	
	Higher vs primary	8.64		3.31-22.57		<.001	
	Vocational vs primary	0.68		0.29-1.64		.39	
**Employment**							
	Part-time vs full-time	0.25		0.11-0.55		.001	
	Pension vs full-time	0.18		0.11-0.29		<.001	
	Never worked vs full-time	0.49		0.18-1.36		.17	
**Marital status**							
	Divorced vs married	0.91		0.46-1.79		.78	
	Miss or bachelor vs married	2.31		1.23-4.34		.01	
	Widow or widower vs married	0.12		0.05-0.31		<.001	
**Residence**							
	City of up to 10,000 vs countryside	0.96		0.57-1.60		.87	
	City of up to 100,000 vs countryside	3.47		1.68-7.18		.001	
	City of over 100,000 vs countryside	5.59		2.72-11.48		<.001	
**MS course**							
	Stable without relapse vs with relapse	1.50		0.87-2.57		.14	
	Progressive vs with relapse	0.47		0.29-0.75		.002	
**Mobility**							
	Independently with devices vs without help	0.53		0.31-0.89		.02	
	With help vs without help	0.18		0.10-0.33		<.001	

^a^MS: multiple sclerosis.

**Table 6:** Credibility of websites dealing with multiple sclerosis.

Internet website	n	Average credibility (0-10)
MS^a^ communities’ websites	153	7.25
Blogs written by patients	151	6.26
MS services’ websites	147	5.47
Doctors’ blogs or websites	130	6.37
Foreign websites	115	6.65

^a^MS: multiple sclerosis.

## Discussion

An examination of how MS patients use the internet may result in a range of observations and conclusions, as shown in previous findings [4,5,6,21]. Our multicenter study confirmed that discovering the preferences of Polish MS patients in connection to internet usage has many practical implications.

As the study revealed, the way Polish individuals suffering from MS use the internet does not depend on their sex but on their age. Similar dependency was also found in the case of the general Polish population [22].

Our study shows that patients with shorter disease duration tend to seek information on the internet more often, which confirms the conclusions of studies on the importance of the information presented to newly diagnosed patients with MS [23,24]. Although the internet was a significant source for obtaining MS-related information, most participants did not find this information suitable for discussion with their doctors, which may stem from the uncertainty connected with treatment methods, the course of the disease, and its possible outcomes. Lack of communication involving the information found on the internet might constitute a potential harmful factor for the patient-doctor relationship.

The accuracy and reliability of health information introduced on the internet and its impact on health care have been frequently discussed [5,6,7,9]. The majority of the examined patients claimed that they pay attention to the information source and would rate its credibility. Websites belonging to MS communities were deemed the most reliable—7.25 on a scale of 0-10, which underlines the importance of discussing the information found online with a medical professional, as well as the significance of the subject on patient-doctor relationships and impact on adherence [21,25].

Cyberpsychological studies are helpful in evaluating whether more engaged patients see medical improvement stemming from deeper adherence and a better understanding of preventive examinations. It is known that the availability of information changes the patients’ expectations in the area of decision making during the treatment [26,27]. All medical treatments, including those for MS, become a cooperative process led by both doctors and their patients. The latter is given a chance to engage and actively participate in choices regarding their health and condition [28].

The internet also provides a new field for innovative forms of psychological help and control of medical suggestions (telemedicine). Auto-diagnosis, including that for MS, is a separate risk connected with the accessibility of the internet, so a person who believes they are suffering from a disease based on information found online should consult a neurologist and follow a doctor’s professional opinion.

MS websites (eg, SMsocialnetwork.com) were perceived by patients to be a useful tool to support health-related coping and social interaction and may suggest a new kind of therapeutic alliance between physicians and people with MS [29].

Building new relationships online has long been a matter of interest for psychologists, who recognize its potential dangers and undeniable advantages facilitating social life for those finding direct communication difficult [4,15,19]. In the case of MS patients, particularly in the later stages of the disease, maintaining contact with others through internet communication from their homes might be a helpful tool for coping with isolation and its negative impact on one’s life [1].

Studies have confirmed that Polish MS patients use the internet to build new relationships, which usually remain outside of the direct communication zone. This might be caused by specific methods of taking care of oneself and one’s family in the face of illness [26]. A similar examination conducted on bigger groups of MS patients confirmed that certain Web-based environments might impact one’s standard of living, which is caused mainly by meeting new people and maintaining contact with them [2,3,17].

Our examination of 1045 MS patients resulted in an objective assessment of many aspects of data collection and was helpful in defining the preferred form of websites. The data on internet browsing behaviors, including the frequency and usage of internet services, facilitate the development of websites to be tailored to the needs and habits of the audience. It is important to note that certain MS patients may encounter problems accessing the websites owing to disabilities, disease symptoms, difficulties with vision, cognitive disorders, and impaired memory, as indicated before [12]. Such adjustments might prove supportive and helpful for MS patients who wish to experience modern medicine’s benefits—personalization and active participation in the treatment process. 

Our study, the first of its kind in Poland, describes the relationship between the clinical and demographic factors of MS patients and their approach and methods applied when searching the internet for information. The results suggest that patients are eager to use the internet to learn about their condition. Although the information found online is rarely discussed with doctors, the possibility of maintaining contact between a patient and their doctor may grant the patient better access to credible information, which may have a positive impact on his or her treatment process. The availability of internet data might aid in the improvement and personalization of health care, answering patients’ specific needs and providing information tailored to one’s stage of disease, disability level, employment situation, and computer skills (including the ability to use the internet).

## Abbreviations

MS: multiple sclerosis
